# Interactions between Age and ITN Use Determine the Risk of Febrile Malaria in Children

**DOI:** 10.1371/journal.pone.0008321

**Published:** 2009-12-23

**Authors:** Philip Bejon, Edna Ogada, Norbert Peshu, Kevin Marsh

**Affiliations:** 1 Kenyan Medical Research Institute (KEMRI), Centre for Geographic Medicine Research (Coast), Kilifi, Kenya; 2 Nuffield Department of Medicine, University of Oxford, Oxford, United Kingdom; London School of Hygiene and Tropical Medicine, United Kingdom

## Abstract

**Background:**

Control measures which reduce individual exposure to malaria are expected to reduce disease, but also to eventually reduce immunity. Reassuringly, long term data following community wide ITN distribution show sustained benefits at a population level. However, the more common practice in Sub-Saharan Africa is to target ITN distribution on young children. There are few data on the long term outcomes of this practice.

**Methodology/Principal Findings:**

Episodes of febrile malaria were identified by active surveillance in 383 children over 18 months of follow up. In order to compare the short and long term outcomes of ITN use, we examined interactions between ITN use and age (12–42 months of age versus 42–80 months) in determining the risk of febrile malaria. ITN use and older age protected against the first or only episode of malaria (Hazard Ratio [HR]  = 0.33, 95%CI 0.17–0.65 and HR  = 0.30, 95%CI 0.17–0.51, respectively). The interaction term between ITN use and older age was HR  = 2.91, 95%CI 1.02–8.3, p = 0.045, indicating that ITNs did not protect older children. When multiple episodes were included in analysis, ITN use and older age were again protective against malaria episodes (Incident Rate Ratio [IRR]  = 0.43 95%CI 0.27–0.7) and IRR  = 0.23, 95%CI 0.13–0.42, respectively) and the interaction term indicated that ITNs did not protect older children (IRR  = 2.71, 95%CI 1.3–5.7, p = 0.008).

**Conclusions/Significance:**

These data on age interactions with ITN use suggest that larger scale studies on the long term individual outcomes should be undertaken if the policy of targeted ITN use for vulnerable groups is to continue.

## Introduction

Malaria is a global public health problem. There were over 500 million episodes globally in 2002, and over a million deaths in Africa [1], [2]. The evidence that insecticide treated nets (ITNs) reduce malaria risk and mortality is overwhelming [3]. Recently, ITN distribution has become more widespread in some endemic countries [4] with reductions in childhood mortality [5].

The relationship between malaria transmission and morbidity/mortality is not linear [6], perhaps because acquiring immunity at an earlier age under high transmission conditions offsets the overall increase in life time cases of malaria one might expect to see. Hence, the average age of children with severe malaria increases as transmission falls [7]. This might indicate that ITN use would not lead to sustained reductions in malaria incidence as the effects of waning immunity become apparent. Fortunately, this has not been seen the long term follow up of communities randomized to ITN use in cluster randomized trials [8], [9], [10], despite reductions in some antibody responses with ITN use [11]. Although these trials focused on outcomes in under 5 year old children, the mode of distribution within the clusters was community wide use. The current practice in most malaria endemic countries is to target vulnerable groups in distribution programmes, and this policy may not yield the same long term results since community-wide ITN use reduces mosquito survival and infectivity [12], reducing overall transmission [13], [14], [15]. These reductions in transmission may offset reductions in immunity, explaining the sustained benefits of ITN use [16].

There are few data on the long term effects of individual ITN use. In order to examine the long term outcome of ITN use, we examine the interactions between ITN use and age of the children in observational data from Kilifi district, Kenya. We compare residence in villages at lower transmission intensity with the effect of ITN use.

## Methods

### Study Design

Ethical approval was obtained from the Kenyan Medical Research Institute National Ethics Committee, the Central Oxford Research Ethics Committee, and the London School of Hygiene and Tropical Medicine Ethics Committee. Parents of all children were approached for written informed consent before research began. The data presented here were generated during a randomised, controlled and double blind vaccine trial of an ineffective candidate vaccination to malaria. The details of study design are described elsewhere [17]. Data from extended follow up are included [18]. All children were treated with antimalarials at the start of follow up, using 7 days of directly observed dihydroartemisinin monotherapy (2 mg per kg on the first day, followed by 1 mg per kg for 6 days). Subjects were followed up for a maximum of 18 months, and a cross-sectional venous blood sample was conducted at 3 months.

### Location

The study was carried out in Junju sublocation in Kilifi District, on the Kenyan coast. Junju contains a group of 5 closely related villages within the Chonyi area of Kilifi district. Kilifi is malaria-endemic, with all year round transmission and two high transmission seasons [19]. Transmission intensity is falling in Kilifi District [20], [21], but in Junju sublocation, the parasite prevalence was 71% in 1–7 year old children at the start of the study. At the time of the study (2005), the local dispensary sold subsidised ITNs to parents of young children and pregnant mothers attending the dispensary. Nationally, this policy had increased the coverage of ITNs from 7.1% in 2004 (when commercial retail was the main source of nets) to 23.5%. Shortly after the end of the study, in 2006, there was a government initiated mass distribution campaign, which increased ITN coverage to 67.3% [4].

### Participants

The participating children were aged 1–6 years old (inclusive) at enrolment, healthy, and resident in Junju sublocation, Kilifi District. They were recruited following public meetings to invite potential participants.

### Follow Up

Children were visited every week by fieldworkers and their axillary remperature was recorded. When the temperature was greater than 37.5 degrees, a blood film was made and a rapid near-patient test for malaria conducted. If the rapid test was positive, a full course of artmether/lumefantrine was given. Where the axillary temperature was less than 37.5 degrees, but the parents reported a fever, the fieldworkers returned three times over 24hours to take repeated measurements. If the child was febrile, a blood film and rapid test were taken. Parents could bring their children for assessment at any time, and the fieldworkers were resident in the study area. Cross-sectional blood films were performed on all children after 3 months of follow up.

Bednet use was assessed by fieldworkers visiting the subject's home at the beginning of the study, at 4–5 monthly intervals and at the end of the study. They observed whether the bednet was hung over the child's sleeping space, asked whether it had been recently treated, and examined the number of holes that could admit a fingertip.

### Analysis

Febrile malaria was defined as an axillary temperature greater than 37.5 degrees centigrade, with a Plasmodium falciparum parasitemia greater than 2,500 parasites per µl [19]. Children were considered ITN users if they used ITNs throughout the 18 month period. Non-users were those who did not use an ITN at the beginning or end of the study. Isolated instances of non-use on a single visit were ignored if use was consistent before and after, and vice versa. A sensitivity analysis was conducted to examine the effect of numbers of holes in the ITNs and inclusion of the children who acquired or lost ITNs during the period of study.

Subjects came from 5 villages. Transmission intensity for each village was determined by the rate of first reinfection occurring over three months monitoring following curative anti-malarial therapy (including both febrile and asymptomatic infection). Two villages formed the “high transmission” group (n = 207) and the remaining three villages formed the “low transmission” group (n = 176). Transmission was stratified according to re-infection rates measured during the three months following curative anti-malarials, which were 50% and 49% in the two villages in the “high transmission” group, and 39%, 36% and 27% among the three villages in the low transmission group. The parasite rates prior to curative anti-malarials were 78% and 69% in the high transmission group and 65%, 61% and 43% in the low transmission group [22]. Age was analysed by dividing children into two equally sized groups. The younger group was 12–42 months of age (n = 192) and the older was >42–80 months (n = 191). We reasoned that dividing age into two categories facilitated an analysis of interactions between age/ITN use and age/transmission intensity. An analysis using age as a continuous variable was also conducted for comparison.

Analysis of the 18 month follow up was done using survival models for the first or only episode of febrile malaria (Cox regression) and on the total number of episodes (Poisson regression). Analysis of ITN use was adjusted by village, analysis of transmission intensity was adjusted by ITN use, and analysis of the total number of episodes was adjusted by time at risk. The survival plots were not adjusted.

Further analysis of the first 3 months of follow up was done using a categorisation of outcome to distinguish immunity from exposure, as described previously [22]. Children were assigned one of three categories; febrile malaria (i.e. one or more episode of febrile malaria during the 3 months), acquiring asymptomatic infection (i.e. no detected episode of febrile malaria but asymptomatic parasitaemia was identified as the cross-sectional bleed after 3 months), or uninfected (no episode of febrile malaria was identified and the 3 month cross-sectional sample showed no parasites). Logistic regression models were then fitted for two comparisons; febrile malaria vs asymptomatic malaria, and any infection (i.e. febrile plus asymptomatic) vs uninfected status. We used STATA version 10 (StataCorp, Texas) for all analyses.

## Results

**Figure 1:**
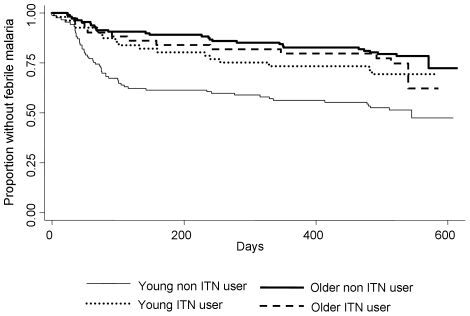
Survival plot of time to first episode of febrile malaria (>2,500 parasites per µl). Children are divided by age (in two categories) and by ITN use. P<0.0001 by logrank.

**Figure 2:**
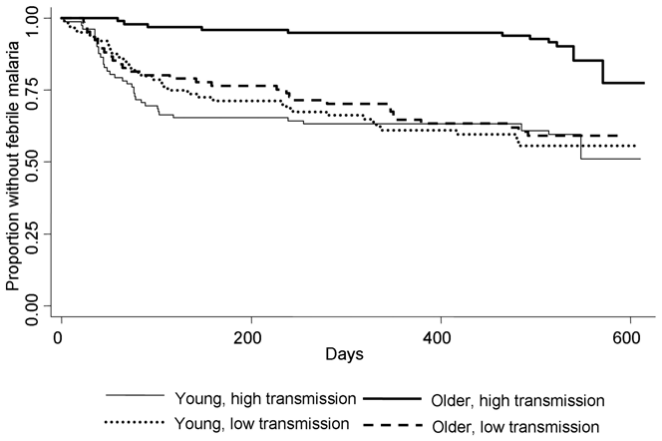
Survival plot of time to first episode of febrile malaria (>2,500 parasites per µl). Children are divided by age (in two categories) and by residence (in two transmission zones). P<0.0001 by logrank.

There were 355 children with data on ITN use. 102 children slept under an ITN, 253 did not. 18 children had nets with more than 3 holes, and 2 had nets with more than 6 holes. 74 children acquired an ITN during the 18 months of follow up, and 7 children had ITNs that developed more than 3 holes over the 18 months. 6 children lost the use of an ITN during the study. 236 episodes of febrile malaria were reported. Survival plots (Figures 1 and 2), incidence rates (Table 1) and hazard ratios and incidence rate ratios (Table 2) are shown for this cohort.

**Table 1:** The incidence rates of febrile malaria by age and ITN use.

Category	Incidence	95%CI	Child Years of observation
Young non ITN users	0.51	0.39–0.67	101.6
Young ITN users	0.27	0.17–0.43	63.14
Old non ITN users	0.17	0.12–0.25	157.4
Old ITN users	0.22	0.12–0.38	55.3
Young high trans	0.41	0.30–0.57	87.4
Young low trans	0.43	0.30–0.60	77.4
Old high trans	0.08	0.05–0.15	134.4
Old low trans	0.37	0.26–0.53	81

**Table 2:** The effect of ITN use and residence at low transmission intensity on risk of febrile malaria, and the interactions with age group.

Category	n	Survival analaysis			Multiple events		
		HR	95% CI	p	IRR	95% CI	p
Younger age	137	1				1	
Older age	138	0.3	0.17–0.51	<0.001	0.23	0.13–0.42	<0.001
No ITN	179	1			1		
ITN use	96	0.33	0.17–0.65	0.001	0.43	0.27–0.7	<0.001
ITN use*Older	–	2.91	1.02–8.3	0.045	2.72	1.3–5.7	0.008
High Trans.	160	1			1		
Low trans.	115	1.34	0.8–2.3	0.31	1.31	0.9–1.9	0.17
Low trans*Older	–	3.1	1.2–8.5	0.025	2.23	1.1–4.5	0.023

Hazard ratios (HR) from Cox regression and incidence rate ratios (IRR) from Poisson regression are shown for survival and multiple event analyses, respectively. Children are divided into equal groups of younger (12–42 months) and older (42–80 months) children. The interaction term for ITN use and older age is shown by *. (i.e. the HR/IRR for what was observed in older children using ITNs compared with what would have been predicted for the additive effect of ITN use and older age).

The survival plots for time to first or only episode of febrile malaria (Figures 1 and 2) show that the effect of ITN and village varies by age group. In younger children, ITNs were protective, but in older children there was a tendency for more febrile malaria with ITN use (Figure 1). An analogous pattern was seen for residence in a low transmission village (Figure 2). In younger children, transmission intensity had no effect on overall malaria risk, but in older children lower transmission was associated with a greater risk of febrile malaria. Thus, the effect of both low transmission intensity and ITN use was modified in the older age group in the same direction. (Table 2).

On Cox regression for risk of first event and Poisson regression for multiple events, the interaction terms for ITN use and older age, and for low transmission and older age, were significantly greater than 1. This indicates an antagonistic interaction (i.e. that the effects of ITN use/low transmission in the older age group counteracted the protection one would normally expect from increasing age alone).

### Sensitivity Analysis

We examined how sensitive the analysis was to different definitions of ITN use. If we did not exclude children who acquired ITNs during the study or whose nets developed more than 6 holes during the study, the interaction term for survival analysis gave HR = 2.19 (95%CI 0.86–5.6, p = 0.10). If the definition of ITN use was a net with less than 3 holes or less than 6 holes the interaction terms were HR = 2.75 (95%CI 0.95–7.95, p = 0.062) and HR = 2.52 (95%CI 0.91–6.98, p = 0.075), respectively. For Poisson analyses of multiple events, without the exclusions for a change in ITN status and for definitions of use based on 3 or 6 holes, the interaction terms were IRR = 3.02 (95%CI 1.56–5.83, p = 0.001), IRR = 3.28 (95%CI 1.52–7.04, p = 0.002) and IRR = 2.83 (95%CI 1.38–5.80, p = 0.004).

When age was used as a continuous variable, the interaction terms on survival analysis were HR = 1.25 per year (95%CI 0.9–1.7, p = 0.16) and 1.55 (1.2–2.1, p = 0.003) for the effect of ITN use and village respectively. The interaction terms on Poisson analysis for multiple events were IRR = 1.2 (95%CI 0.9–1.5, p = 0.19), IRR = 1.3(95%CI 1.1–1.6, p = 0.012) for ITN use and village, respectively. However, the goodness of fit by log likelihood was lower for age as a continuous variable compared with age as a categorical variable (log likelihood = −603.3 vs −601.6 for Cox regression and log likelihood = −401.3 vs −400.8 for Poisson regression). Furthermore, the residuals were unevenly distributed for age as a continuous variable (p = 0.001). Because of this non-linear effect of age on malaria risk, we preferred analyses that used age as a categorical variable (as given above).

### An Analysis to Distinguish Exposure and Immunity

In order to examine the contributions of exposure and immunity to the altered effect of ITNs and transmission intensity, we examined odds ratios for febrile malaria versus asymptomatic parasitaemia (Table 3). Age was associated with a reduced risk of febrile malaria compared with asymptomatic parasitaemia. The significant interaction terms demonstrate that the effects of ITN use and of low transmission intensity on febrile malaria vs asymptomatic parasitaemia varied by age. By contrast, ITN use and low transmission intensity are associated with a reduced risk of infection (i.e. combining asymptomatic parasitaemia and febrile malaria) compared with uninfected status, and this effect did not vary by age. Hence, the protective effect of ITN use or low transmission intensity against infection per se did not vary by age, but the risk that infection is associated with a febrile episode did.

**Table 3:** Logistic models to examine the effect of ITN use and transmission intensity on the category of malaria infection, and their interactions with age.

	Febrile vs. Asymptomatic	Infected vs. uninfected			
	n	OR	CI	p	n	OR	CI	p
Younger Age	74	1			179	1		
Older Age	77	0.08	0.02–0.25	<0.001	180	0.62	0.3–1.1	0.13
No ITN	118	1			262	1		
ITN use	32	0.26	0.08–0.88	0.031	94	0.52	0.3–1.1	0.07
ITN use* Old		6.5	1.1–38	0.04	–	1.6	0.6–4.4	0.35
High trans	96	1			194	1		
Low trans.	55	2.2	0.75–6.6	0.15	165	0.32	0.2–0.6	<0.001
Low trans* Old	–	4.2	0.85–20.1	0.08	–	2.5	1.0–5.9	0.043

Odds ratios (OR) from logistic regression are shown, for the risk of febrile malaria compared with asymptomatic infection, and then for the risk of any malaria infection (i.e. asymptomatic infection or febrile malaria) with uninfected status. Children are divided into equal groups of younger (12–42 months) and older (42–80 months) children. The ORs are shown for the effect of ITN use according among the younger and then older children separately, and then the interaction term for ITN use and older age is shown by *. The same format is then used for the effect of residence at low transmission intensity (Low trans.).

## Discussion

We show that residence in a village at lower transmission intensity results in a relative increase in the risk of febrile malaria in older children. This is consistent with previous comparisons of different regions and countries within Sub-Saharan Africa [6], [7], and the pattern of severe disease seen at different altitudes [23]. The obvious conclusion might be that ITN use would have the same effect, so causing concern about the overall outcome of ITN distribution programmes. In vitro markers of blood stage immunity are reduced in some [24], but not all [11], studies comparing ITN users with non-users. Children on prolonged chemoprophylaxis for malaria have reduced immunity to malaria, and consequently higher rates of febrile malaria over long term follow up [25]. Our data showing that ITN use reduced febrile malaria risk in younger but not older children is consistent with older children having used ITNs when they were younger, and therefore having acquired less immunity.

However, our data should not be interpreted in isolation. Long term follow up following randomized trials of ITN use have consistently shown sustained reductions in morbidity and mortality [8], [9], [10]. In these trials, geographical clusters were randomized to community wide distribution of ITNs. The use of ITNs in this way reduces overall transmission by reducing the survival and infectivity of mosquitoes [12], and mathematical models suggest falling transmission counteracts the effects of reduced immunity [16]. Other models suggest that where a minority of the community uses ITNs, there is little overall fall in transmission [26]. Among the children we studied, only a minority used ITNs (in contrast to the situation following community wide distribution). Hence, falling overall transmission may not have been present to oppose the effects of reduced immunity in older children. Hence, our data do not contradict those showing continued reductions in malaria rates continue to fall following community wide distribution. Rather, they reinforce the benefits of community wide ITN distribution over targeting vulnerable groups.

Our study was observational, and competing explanations for the apparent reduction in efficacy of ITNs in older children should be considered: First, parents of older children might buy ITNs if they notice their child is at high risk of malaria, but parents of younger children have not yet noticed that their child is at high risk. Hence the child's risk determines ITN use in older children, but not in younger. This seems unlikely since there was no evidence that ITN use was significantly more common among older children in the higher transmission villages (29% compared with 28% in low transmission villages, p = 0.17), and ITN use did not increase among older children (26% compared with 31% in younger children).

Second, we have assumed that the older ITN users were long-term ITN users. Therefore, an increased risk of malaria can be explained by having not acquired immunity during a prolonged period of ITN use. In fact, some older children may have recently started using an ITN. This would not explain our results, however, since we would predict that older children who recently acquired an ITN would have the immunity of non-users, but the lowered exposure of an ITN user, and hence much lower than expected rates of malaria. Hence, this would increase the apparent protective efficacy of an ITN. Conversely, it is possible that some older ITN non-users were previous ITN users, but passed the ITNs on to younger siblings. We would predict that such children would have the lower immunity of ITN users, but the higher exposure of ITN non-users, and much higher rates of malaria. Hence, this would also increase the apparent protective efficacy of an ITN. As it happened, we found higher than expected rates of malaria among older children who were ITN users.

Third, it is possible that the recently distributed ITNs were of better quality than those distributed several years previously. However, the state of repair of the ITNs was documented by fieldworker visits, and was generally good. Furthermore, the results were not sensitive to definitions of ITN use based on number of holes observed in the net. The pattern of HR and IRRs was similar with the varying definitions. Significance was variable on survival analysis, but consistent for multiple episodes.

Fourth, it may be that ITNs are less effective in older children because of behavioural differences (e.g. they go to sleep later, after being bitten by mosquitoes). Studies in Cambodia have suggested that older children are not protected when randomized to ITN use [27]. However, the *Anopheles dirus* complex of South East Asia bites earlier in the evening than the common African vectors [28], and interactions between ITN use and age have not previously been seen in children in Africa recently randomized to ITNs [29], [30]. In fact, efficacy of ITNs among adolescents [31] and adults [32] in Africa has been described.

Fifth, use of ITNs by other homestead members is protective. If use by other homestead members was associated with the age of the ITN using child, this could explain our results. However, the majority of homesteads in our study (82%) contained both older and younger children, so this explanation is unlikely. Finally, it is unclear what effect clearing infection at the start of the study may have had. It is possible the chronic parasitaemia helps to maintain immunity (i.e. premunition), but there was no evidence of an early increase in malaria cases among older children in the survival plots. In any case, it is not obvious how the effect of clearing parasites could be different for older ITN users compared with younger ITN users.

Other studies have demonstrated that ITNs protect against parasitaemia in all age groups [33]. Here, we have described a reduced efficacy of ITNs in older children for febrile episodes. When we analysed asymptomatic parasitaemia as well as febrile episodes, we found that ITN use continued to protect against malaria infection per se in older children, but was associated with a shift from asymptomatic parasitaemia to febrile malaria in older children. Taken together, this suggests that ITNs continue to protect against mosquito bites, but are associated with a higher likelihood that parasitisation will cause fever in older children. This conclusion fits well with other epidemiological observations [6], [7], [23] and our current understanding of blood stage immunity [25], [34].

Policy may move towards community wide ITN distribution programmes rather than continue targeting vulnerable populations, to ensure that reduced transmission offsets reductions in host immunity [16]. However, if the policy of targeted distribution for under 5 year old children continues, then large scale, long term studies should monitor the outcomes in terms of individual susceptibility.

## References

[1] Snow RW, Guerra CA, Noor AM, Myint HY, Hay SI (2005). The global distribution of clinical episodes of Plasmodium falciparum malaria.. Nature.

[2] Snow RW, Craig M, Deichmann U, Marsh K (1999). Estimating mortality, morbidity and disability due to malaria among Africa's non-pregnant population.. Bull World Health Organ.

[3] Lengeler C (2004). Insecticide-treated bednets and curtains for preventing malaria (Cochrane Review).. Cochrane Database Syst Rev.

[4] Noor AM, Amin AA, Akhwale WS, Snow RW (2007). Increasing coverage and decreasing inequity in insecticide-treated bed net use among rural Kenyan children.. PLoS Med.

[5] Fegan GW, Noor AM, Akhwale WS, Cousens S, Snow RW (2007). Effect of expanded insecticide-treated bednet coverage on child survival in rural Kenya: a longitudinal study.. Lancet.

[6] Snow RW, Omumbo JA, Lowe B, Molyneux CS, Obiero JO (1997). Relation between severe malaria morbidity in children and level of Plasmodium falciparum transmission in Africa.. Lancet.

[7] Okiro EA, Al-Taiar A, Reyburn H, Idro R, Berkley JA (2009). Age patterns of severe paediatric malaria and their relationship to Plasmodium falciparum transmission intensity.. Malar J.

[8] Lindblade KA, Eisele TP, Gimnig JE, Alaii JA, Odhiambo F (2004). Sustainability of reductions in malaria transmission and infant mortality in western Kenya with use of insecticide-treated bednets: 4 to 6 years of follow-up.. Jama.

[9] Binka FN, Hodgson A, Adjuik M, Smith T (2002). Mortality in a seven-and-a-half-year follow-up of a trial of insecticide-treated mosquito nets in Ghana.. Trans R Soc Trop Med Hyg.

[10] Diallo DA, Cousens SN, Cuzin-Ouattara N, Nebie I, Ilboudo-Sanogo E (2004). Child mortality in a West African population protected with insecticide-treated curtains for a period of up to 6 years.. Bull World Health Organ.

[11] Kariuki SK, Lal AA, Terlouw DJ, ter Kuile FO, Ong'echa JM (2003). Effects of permethrin-treated bed nets on immunity to malaria in western Kenya II. Antibody responses in young children in an area of intense malaria transmission.. Am J Trop Med Hyg.

[12] Gimnig JE, Kolczak MS, Hightower AW, Vulule JM, Schoute E (2003). Effect of permethrin-treated bed nets on the spatial distribution of malaria vectors in western Kenya.. Am J Trop Med Hyg.

[13] Binka FN, Indome F, Smith T (1998). Impact of spatial distribution of permethrin-impregnated bed nets on child mortality in rural northern Ghana.. Am J Trop Med Hyg.

[14] Howard SC, Omumbo J, Nevill C, Some ES, Donnelly CA (2000). Evidence for a mass community effect of insecticide-treated bednets on the incidence of malaria on the Kenyan coast.. Trans R Soc Trop Med Hyg.

[15] Hawley WA, Phillips-Howard PA, ter Kuile FO, Terlouw DJ, Vulule JM (2003). Community-wide effects of permethrin-treated bed nets on child mortality and malaria morbidity in western Kenya.. Am J Trop Med Hyg.

[16] Gosling RD, Ghani AC, Deen JL, von Seidlein L, Greenwood BM (2008). Can changes in malaria transmission intensity explain prolonged protection and contribute to high protective efficacy of intermittent preventive treatment for malaria in infants?. Malar J.

[17] Bejon P, Mwacharo J, Kai O, Mwangi T, Milligan P (2006). A Phase 2b Randomised Trial of the Candidate Malaria Vaccines FP9 ME-TRAP and MVA ME-TRAP among Children in Kenya.. PLoS Clin Trials.

[18] Bejon P, Ogada E, Mwangi T, Milligan P, Lang T (2007). Extended follow-up following a phase 2b randomized trial of the candidate malaria vaccines FP9 ME-TRAP and MVA ME-TRAP among children in Kenya.. PLoS ONE.

[19] Mwangi TW, Ross A, Snow RW, Marsh K (2005). Case definitions of clinical malaria under different transmission conditions in Kilifi District, Kenya.. J Infect Dis.

[20] O'Meara WP, Mwangi TW, Williams TN, McKenzie FE, Snow RW (2008). Relationship between exposure, clinical malaria, and age in an area of changing transmission intensity.. Am J Trop Med Hyg.

[21] Okiro EA, Hay SI, Gikandi PW, Sharif SK, Noor AM (2007). The decline in paediatric malaria admissions on the coast of Kenya.. Malar J.

[22] Bejon P, Warimwe G, Mackintosh CL, Mackinnon MJ, Kinyanjui SM (2009). Immunity to febrile malaria in children: an analysis that distinguishes immunity from lack of exposure.. Infect Immun.

[23] Reyburn H, Mbatia R, Drakeley C, Bruce J, Carneiro I (2005). Association of transmission intensity and age with clinical manifestations and case fatality of severe Plasmodium falciparum malaria.. Jama.

[24] Askjaer N, Maxwell C, Chambo W, Staalsoe T, Nielsen M (2001). Insecticide-treated bed nets reduce plasma antibody levels and limit the repertoire of antibodies to Plasmodium falciparum variant surface antigens.. Clin Diagn Lab Immunol.

[25] Aponte JJ, Menendez C, Schellenberg D, Kahigwa E, Mshinda H (2007). Age interactions in the development of naturally acquired immunity to Plasmodium falciparum and its clinical presentation.. PLoS Med.

[26] Killeen GF, Smith TA, Ferguson HM, Mshinda H, Abdulla S (2007). Preventing childhood malaria in Africa by protecting adults from mosquitoes with insecticide-treated nets.. PLoS Med.

[27] Sochantha T, Hewitt S, Nguon C, Okell L, Alexander N (2006). Insecticide-treated bednets for the prevention of Plasmodium falciparum malaria in Cambodia: a cluster-randomized trial.. Trop Med Int Health.

[28] Obsomer V, Defourny P, Coosemans M (2007). The Anopheles dirus complex: spatial distribution and environmental drivers.. Malar J.

[29] Phillips-Howard PA, Nahlen BL, Kolczak MS, Hightower AW, ter Kuile FO (2003). Efficacy of permethrin-treated bed nets in the prevention of mortality in young children in an area of high perennial malaria transmission in western Kenya.. Am J Trop Med Hyg.

[30] ter Kuile FO, Terlouw DJ, Phillips-Howard PA, Hawley WA, Friedman JF (2003). Impact of permethrin-treated bed nets on malaria and all-cause morbidity in young children in an area of intense perennial malaria transmission in western Kenya: cross-sectional survey.. Am J Trop Med Hyg.

[31] Leenstra T, Phillips-Howard PA, Kariuki SK, Hawley WA, Alaii JA (2003). Permethrin-treated bed nets in the prevention of malaria and anemia in adolescent schoolgirls in western Kenya.. Am J Trop Med Hyg.

[32] ter Kuile FO, Terlouw DJ, Phillips-Howard PA, Hawley WA, Friedman JF (2003). Reduction of malaria during pregnancy by permethrin-treated bed nets in an area of intense perennial malaria transmission in western Kenya.. Am J Trop Med Hyg.

[33] Noor AM, Moloney G, Borle M, Fegan GW, Shewchuk T (2008). The use of mosquito nets and the prevalence of Plasmodium falciparum infection in rural South Central Somalia.. PLoS ONE.

[34] Langhorne J, Ndungu FM, Sponaas AM, Marsh K (2008). Immunity to malaria: more questions than answers.. Nat Immunol.

