# Correction: Interactions between Age and ITN Use Determine the Risk of Febrile Malaria in Children

**DOI:** 10.1371/annotation/73a096b6-934c-4bc7-b0b1-de1f11231350

**Published:** 2011-02-17

**Authors:** Philip Bejon, Edna Ogada, Norbert Peshu, Kevin Marsh

The funding information for PB is incorrect. The correct funding information for PB is: "P. Bejon is supported by the NIHR Biomedical Research Centre Oxford."

